# Gender Disparity in Evaluation of Internal Medicine Clerkship Performance

**DOI:** 10.1001/jamanetworkopen.2021.15661

**Published:** 2021-07-02

**Authors:** Deborah J. Gorth, Rogan G. Magee, Sarah E. Rosenberg, Nina Mingioni

**Affiliations:** 1 Sidney Kimmel Medical College at Thomas Jefferson University, Philadelphia, Pennsylvania; 2 Department of Medicine, Sidney Kimmel Medical College at Thomas Jefferson University, Philadelphia, Pennsylvania

## Abstract

**Importance:**

Women studying medicine currently equal men in number, but evidence suggests that men and women might not be evaluated equally throughout their education.

**Objective:**

To examine whether there are differences associated with gender in either objective or subjective evaluations of medical students in an internal medicine clerkship.

**Design, Setting, and Participants:**

This single-center retrospective cohort study evaluated data from 277 third-year medical students completing internal medicine clerkships in the 2017 to 2018 academic year at an academic hospital and its affiliates in Pennsylvania. Data were analyzed from September to November 2020.

**Exposure:**

Gender, presumed based on pronouns used in evaluations.

**Main Outcomes and Measures:**

Likert scale evaluations of clinical skills, standardized examination scores, and written evaluations were analyzed. Univariate and multivariate linear regression were used to observe trends in measures. Word embeddings were analyzed for narrative evaluations.

**Results:**

Analyses of 277 third-year medical students completing an internal medicine clerkship (140 women [51%] with a mean [SD] age of 25.5 [2.3] years and 137 [49%] presumed men with a mean [SD] age of 25.9 [2.7] years) detected no difference in final grade distribution. However, women outperformed men in 5 of 8 domains of clinical performance, including patient interaction (difference, 0.07 [95% CI, 0.04-0.13]), growth mindset (difference, 0.08 [95% CI, 0.01-0.11]), communication (difference, 0.05 [95% CI, 0-0.12]), compassion (difference, 0.125 [95% CI, 0.03-0.11]), and professionalism (difference, 0.07 [95% CI, 0-0.11]). With no difference in examination scores or subjective knowledge evaluation, there was a positive correlation between these variables for both genders (women: *r* = 0.35; men: *r* = 0.26) but different elevations for the line of best fit (*P* < .001). Multivariate regression analyses revealed associations between final grade and patient interaction (women: coefficient, 6.64 [95% CI, 2.16-11.12]; *P* = .004; men: coefficient, 7.11 [95% CI, 2.94-11.28]; *P* < .001), subjective knowledge evaluation (women: coefficient, 6.66 [95% CI, 3.87-9.45]; *P* < .001; men: coefficient, 5.45 [95% CI, 2.43-8.43]; *P* < .001), reported time spent with the student (women: coefficient, 5.35 [95% CI, 2.62-8.08]; *P* < .001; men: coefficient, 3.65 [95% CI, 0.83-6.47]; *P* = .01), and communication (women: coefficient, 6.32 [95% CI, 3.12-9.51]; *P* < .001; men: coefficient, 4.21 [95% CI, 0.92-7.49]; *P* = .01). The model based on the men’s data also included growth mindset as a significant variable (coefficient, 4.09 [95% CI, 0.67-7.50]; *P* = .02). For narrative evaluations, words in context with “he or him” and “she or her” differed, with agentic terms used in descriptions of men and personality descriptors used more often for women.

**Conclusions and Relevance:**

Despite no difference in final grade, women scored higher than men on various domains of clinical performance, and performance in these domains was associated with evaluators’ suggested final grade. The content of narrative evaluations significantly differed by student gender. This work supports the hypothesis that how students are evaluated in clinical clerkships is associated with gender.

## Key Points

**Question:**

Are objective and subjective evaluations of men and women participating in third-year internal medicine clerkships significantly associated with gender?

**Findings:**

In this cohort study of 277 students from a single academic center, women received higher scores for a majority of the evaluated domains of clinical performance, but there was no difference associated with gender in final grade. In addition, the content of narrative evaluations was significantly associated with the gender of the student being evaluated.

**Meaning:**

These findings suggest that students of different genders might not be evaluated equally during internal medicine clerkships.

## Teaser

This cohort study assesses whether there are gender differences in objective or subjective evaluations of third-year medical students participating in an internal medicine clerkship.

## Introduction

In 1966, only 6.9% of medical school graduates were women; more recently, women made up approximately half of medical school graduates.^1^ This shift happened during the lifetimes of half of currently practicing physicians.^2^ During clerkships, medical students are evaluated not only on their medical knowledge but also on how well they assume the role of physician. With that role being historically gendered, implicit bias regarding how a physician should behave may be associated with how medical students are evaluated. Clinical performance evaluations account for the largest portion of clerkship grades, which carry significant weight in residency recruitment.^3,4^ Understanding potential gender-associated differences in clinical evaluation is necessary to ensure equity in the house staff selection process.

Published in 1978, one of the original studies exploring the association of gender with student performance found no difference between men and women in terms of course grades, clinical performance, and both written and oral examinations.^5^ Since that time, additional work has examined the association between gender and medical school performance with mixed results; generally, either no gender-associated difference is observed or a small increase in clinical performance is noted for women.^6,7,8,9,10^ In addition, research has found that narrative evaluations of women include more personality terms, whereas the focus for men includes more competency-related skills.^11,12,13^ These existing studies focus on 1 metric of evaluation, either overall scores or language analysis, without considering individual components of evaluations and how these metrics interact. However, whether differences in narrative evaluations may be traced to differences in clinical performance has not been studied.

We sought to examine how well individual components of clinical evaluations correlate with overall grade and whether that association is preserved when men and women are considered separately. We gathered both quantitative and qualitative clerkship evaluation data from a single school and class year and used regression analysis and natural language processing to examine gender-specific differences in these evaluations. We compared overall grades, the median scores in various domains of clinical performance for men and women, and the associations between these components to determine whether gender-associated differences were observable. We investigated potential differences in how subjective knowledge evaluation may be associated with multiple-choice examination performance. Finally, we analyzed the language in the narrative portion of the clerkship evaluations, attempting to uncover any significant differences in phrasing or word choice. To our knowledge, our study is the first to comprehensively examine the role that gender may play in clerkship evaluation, by investigating the association of gender with the interplay between overall evaluation, examination scores, and the content of narrative evaluations.

## Methods

### Setting, Participants, and Data

This was a single-center retrospective cohort analysis of evaluation data, including clerkship evaluations and the National Board of Medical Examiners Medicine Subject Examination (NBME MSE) scores, of third-year medical students in the 2017 to 2018 academic year at the Sidney Kimmel Medical College (SKMC) of Thomas Jefferson University, Philadelphia, Pennsylvania. The medicine clerkship consists of 8 weeks with inpatient services, 4 weeks at the Thomas Jefferson University Hospital and 4 weeks at an affiliated community academic medical center. Similar to most institutions,^3^ SMKC uses a summative assessment of clinical performance by faculty and house staff, collected and aggregated by an online platform, as well as scores on the NBME MSE, which is taken at the end of the rotation. This study followed the Strengthening the Reporting of Observational Studies in Epidemiology (STROBE) reporting guideline. This work was deemed exempt from review and from the need to obtain informed consent by the institutional review board of Thomas Jefferson University because data were collected as a part of the established curriculum and anonymized prior to analysis. No one received compensation or was offered any incentive for participating in this study.

The clinical performance of students is assessed using the Clerkship Evaluation Form, which consists of a free-text narrative portion, with a prompt to consider the students’ performance with the established Reporter-Interpreter-Manager-Educator model of evaluation,^14^ a Likert scale evaluation (1 = below expectations, 2 = expected, and 3 = exceeds expectations) of various domains in clinical practice (Box), and a suggested final grade (1 = fail, 2 = marginal, 3 = good−, 4 = good, 5 = good+, 6 = excellent, and 7 = honors). In addition, the evaluators state how much time they spent with the student (1 = superficial contact with student or minimal ability to assess this student, 2 = enough time to generally evaluate, 3 = solid amount of time or feel very comfortable about my ability to assess this student). The final clerkship grade consists of the weighted sum of the suggested final grade (70%), the NBME MSE score (10%), and timely completion of projects (20%). All narrative comments and their association with the final suggested grade are reviewed by a grading committee composed of the clerkship director and clerkship site directors from all affiliate sites. Our data set included anonymized student composite evaluations; gender was presumed by examining pronouns.

Box. **Language of Evaluation Prompts Targeting Various Domains of Clinical Performance**

Evaluations included the following prompts along with a Likert scale score (1 = below expectations, 2 = expected, and 3 = exceeds expectations).
Patient interaction
  Ability to establish humanistic rapport with patient.
  Ability to gather essential and accurate information about patients and their conditions through history-taking and physical examination.
Subjective knowledge
  Demonstrates appropriate knowledge base and understanding of diseases.
  Uses evidence-based medicine.
  Applies knowledge in clinical situations and constructs a differential diagnosis.
  Formulates a treatment plan.
Growth mindset
  Able to identify own strengths and areas for improvement.
  Able to accept feedback and incorporate it into daily practice of medicine to improve own performance.
Communication
  Able to communicate with team about clinical, administrative, and personal tasks.
  Ability to report data in both oral and written form in clear, succinct, and organized manner.
  Able to maintain a clear, legible, and appropriate medical record.
  Able to engage patients in education.
Compassion
  Able to demonstrate compassion, integrity, and respect for others.
  Demonstrates sensitivity and responsiveness to a diverse patient population.
  Demonstrates integrity and commitment to ethical principles.
  Respects patient confidentiality.
Resource utilization
  Able to effectively utilize available resources.
  Advocates for patient safety.
  Aware of concepts of cost, quality, and patient safety.
Teamwork
  Works with other health professionals and staff to establish and maintain a climate of mutual respect.
Professionalism
  Demonstrates personal accountability.
  Manages competing needs of personal and professional responsibility.
  Demonstrates trustworthiness to one’s colleagues regarding the care of patients.

### Regression Analysis

Univariate linear regression was used to assess the association between individual domains of clinical performance and the overall median score given by evaluators. Correlation was assessed (Pearson) and significance determined using analysis of covariance (GraphPad). Multivariate linear regression was used to assess the relative importance of each domain to overall evaluation using the lmg method and relaimpo package in RStudio.^15^ This method has been used, for example, in medical education research to attempt to predict clinical performance based on undergraduate records.^16^ Relative importance uses modeling to determine which variables—in our case, score in each individual domain in clinical practice—are more important when determining an outcome—the overall suggested score in this study.

### Natural Language Processing

*Natural language processing* is an umbrella term for a number of quantitative, machine learning approaches to automated analysis of written documents. Word embeddings use a statistical model to describe how often individual words are used together in a context. This analysis generates numeric vectors that represent the relationships between words and phrases throughout the entire text under consideration.^17^ These representations allow for words to be arranged such that the distance between individual vectors represents their typical context; the smaller the distance between words, the more often they are used in context with each other. For example, in calculated word embeddings from a biology textbook, the words *frog* and *amphibian* might be “closer” in the embedding space than the word *frog* would be to the word *mammal*. A similar method has been applied to examine the language of evaluations used for underrepresented minority students.^11^

Word embedding analysis was conducted by replacing all {xxx}’s that originally replaced the students’ names with the appropriate gendered pronouns (she, her, he, or his). Word embeddings were generated 1000 times using the word2vec and text2vec packages in RStudio. For each word embedding, we then queried the top 50 closest word vectors by the euclidean distance to the words *he*, *she*, *her*, or *his*. These top 50 word vectors represented the 50 closest words in context to the pronouns. We tallied the unique words in each of the 1000 lists. Finally, we performed 2-sided χ^2^ tests on each word to identify which were differentially present in context with the target words (*P* < .05 after Benjamini-Hochberg correction).

### Statistical Analysis

We assessed for normality with the Shapiro-Wilk test and evaluated differences with the Mann-Whitney test for nonparametric continuous data, analysis of covariance with the Kruskal-Wallis test for multiple continuous variables, and the χ^2^ test for categorical variables (GraphPad). Data were analyzed from September to November 2020.

## Results

### Overall Grade Distributions

**Figure 1.**
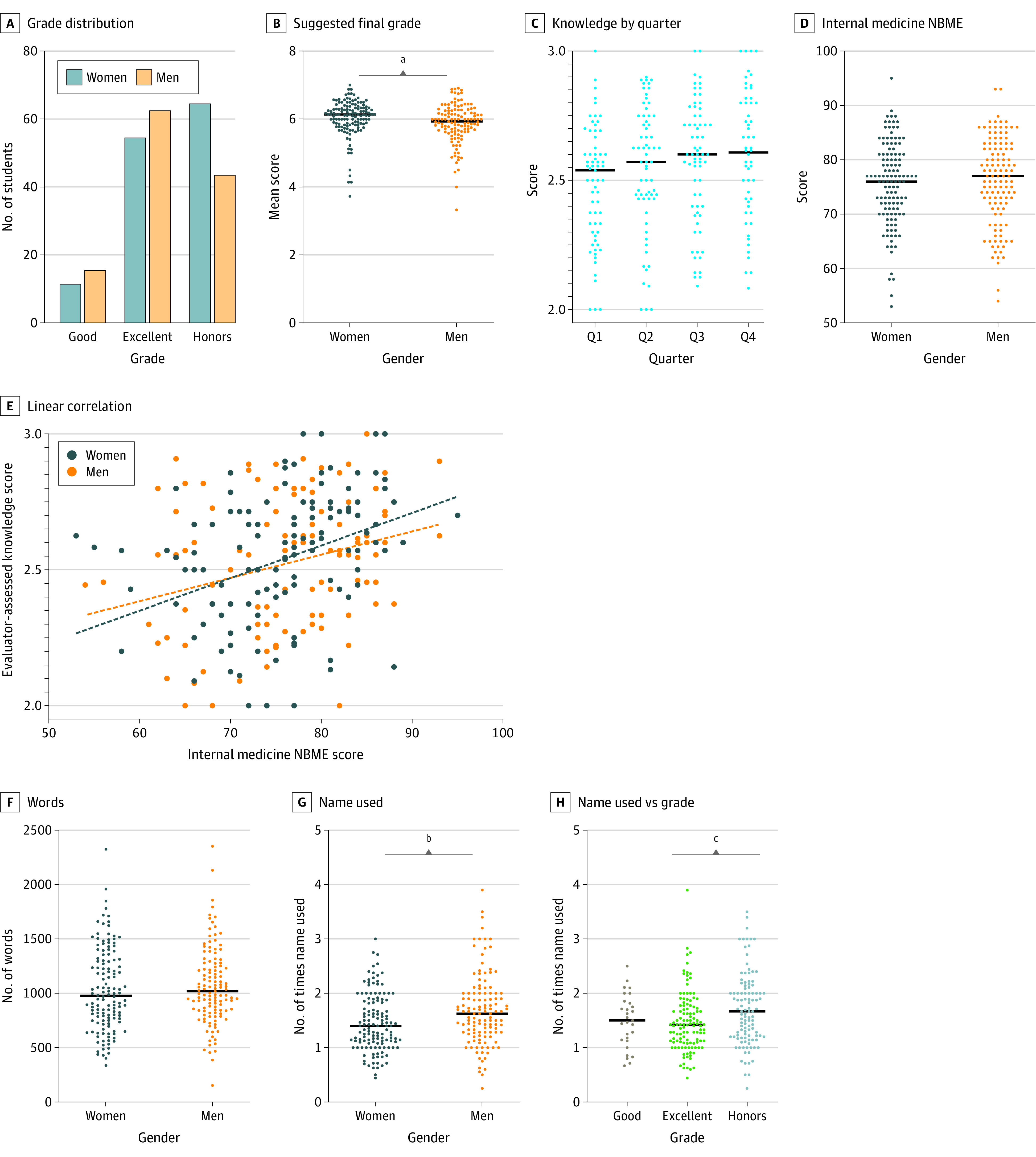
Overall Score Breakdown A, The final overall grade distribution awarded considering final clinical grade and National Board of Medical Examiners (NBME) score; χ^2^ test shows no significant difference. B, The median value of the evaluator-suggested final grade (1 = fail, 2 = marginal, 3 = good−, 4 = good, 5 = good+, 6 = excellent, 7 = honors). C, Subjective knowledge evaluation for each quarter (Q) of the academic year. D, Internal medicine NBME examination score, taken at the end of the clinical rotation. E, Linear correlation between NBME score and the evaluator-assessed demonstrated knowledge base. Positive correlation between NBME performance and subjective knowledge evaluation is significant (women: *r* = 0.35, *P* < .001; men: *r* = 0.26, *P* = .004) for both genders, and the calculated slope of the correlation is not different. However, the y-intercept is significantly lower for women (women: 1.63 [95% CI, 1.29-1.97]; men: 1.87 [95% CI, 1.58-2.17]; *P* < .001); thus, a man with a low NBME score is more likely than a woman with the same score to be rated as having a better knowledge base. F, There was no difference in the number of words used in the narrative evaluation. G, Evaluators were more likely to use men’s names than women’s names when writing narrative evaluations. H, Students who earned honors grades were more likely to have their name mentioned more often in narrative evaluations. ^a^Difference, 0.21 (95% CI, 0.06-0.28); *P* = .003. ^b^Difference, 0.23 (95% CI, 0.08-0.36); *P* = .002. ^c^Difference, 0.24 (95% CI, 0.05-0.42); *P* = .007.

In total, 2589 evaluations of 277 students (140 [51%] presumed women with a mean [SD] age of 25.5 [2.3] years and 137 [49%] presumed men with a mean [SD] age of 25.9 [2.7] years) were collected. There was no difference in final clerkship grade distribution between men and women (Figure 1A). However, women had a higher suggested final score (difference, 0.21 [95% CI, 0.06-0.28]; *P* = .003) (Figure 1B). There was a trend for an increase in subjective knowledge evaluation scores as the academic year progressed, although this trend was not statistically significant (Figure 1C). The NBME MSE scores were not different between genders (Figure 1D). Subjective knowledge evaluation was positively correlated with NBME MSE score for both men and women (women: *r* = 0.35 and *P* < .001; men: *r* = 0.26 and *P* = .004), and the slope was not significantly different by gender. However, the correlation elevation was lower for women (y-intercept for women, 1.63 [95% CI, 1.29-1.97]; y-intercept for men, 1.87 [95% CI, 1.58-2.17]; *P* < .001) (Figure 1E). The length of the narrative comments did not differ between genders (Figure 1F), but evaluators more often referenced men by name (difference, 0.23 [95% CI, 0.08-0.36]; *P* = .002) (Figure 1G). Students receiving a grade of honors were referred to by name more often than students receiving a grade of excellent (*P* = .007) (Figure 1H).

**Figure 2.**
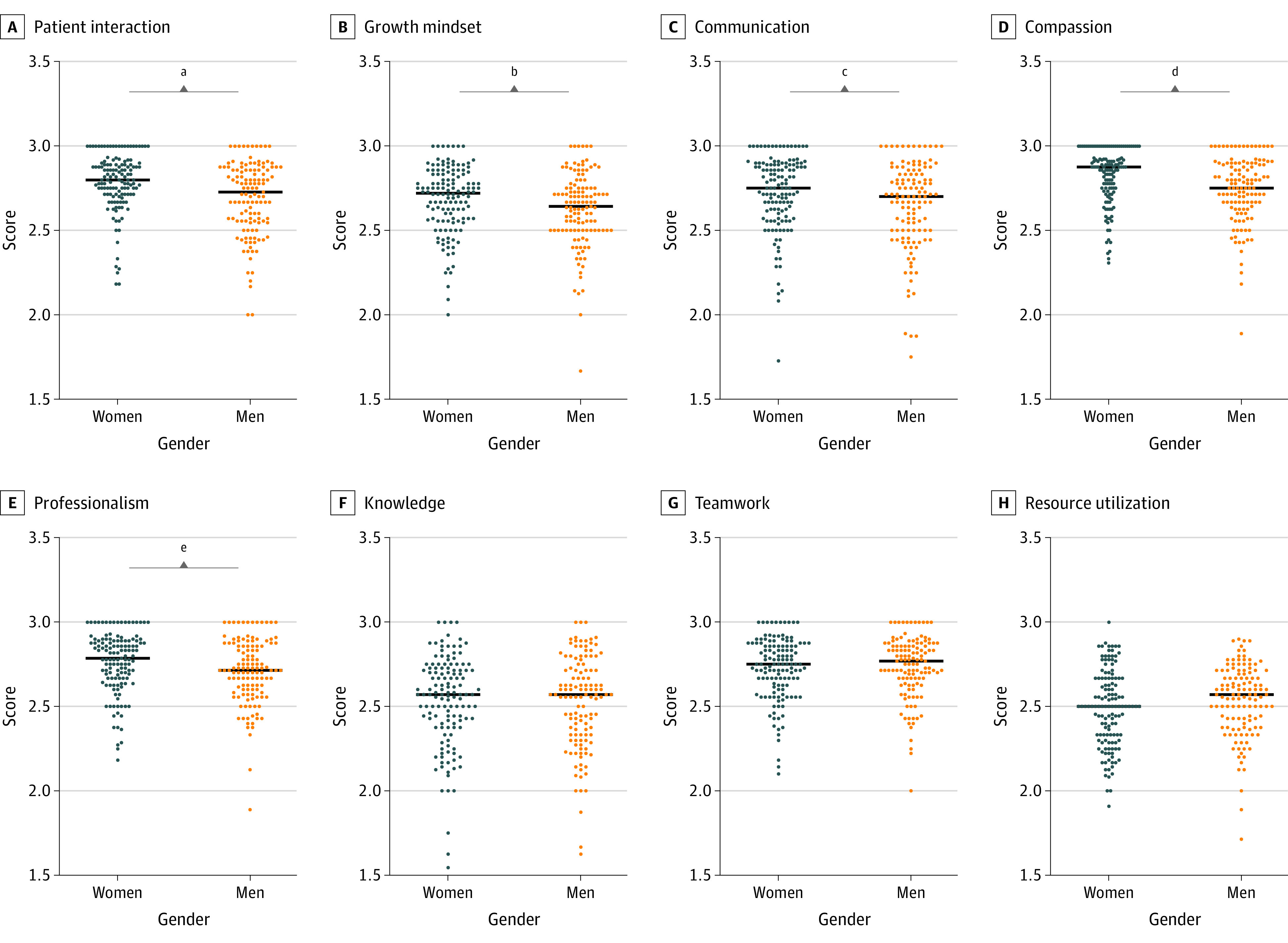
Comparison of the Performance on Each Subcomponent of the Evaluation The mean Likert scale value for each evaluation section (1 = below expectations, 2 = meets expectations, and 3 = exceeds expectations). Women scored significantly higher on the questions targeting their patient-centered performance, growth mindset, communication, compassion, and professionalism. There was no significant gender difference for the remaining questions. ^a^Difference, 0.07 (95% CI, 0.04-0.13); *P* < .001. ^b^Difference, 0.08 (95% CI, 0.01-0.11); *P* = .01. ^c^Difference, 0.05 (95% CI, 0-0.12); *P* = .01. ^d^Difference, 0.13 (95% CI, 0.03-0.11); *P* < .001. ^e^Difference, 0.07 (95% CI, 0-0.11); *P* = .02.

Women received better Likert scale scores on various individual domains of clinical performance, including patient interaction (difference, 0.07 [95% CI, 0.04-0.13]; *P* < .001), growth mindset (difference, 0.08 [95% CI, 0.01-0.11]; *P* = .01), communication (difference, 0.05 [95% CI, 0-0.12]; *P* = .01), compassion (difference, 0.13 [95% CI, 0.03-0.11]; *P* < .001), and professionalism (difference, 0.07 [95% CI, 0-0.11]; *P* = .02) (Figure 2). There was no difference in the subjective knowledge evaluation, teamwork, or resource utilization.

### Regression Analysis

**Figure 3.**
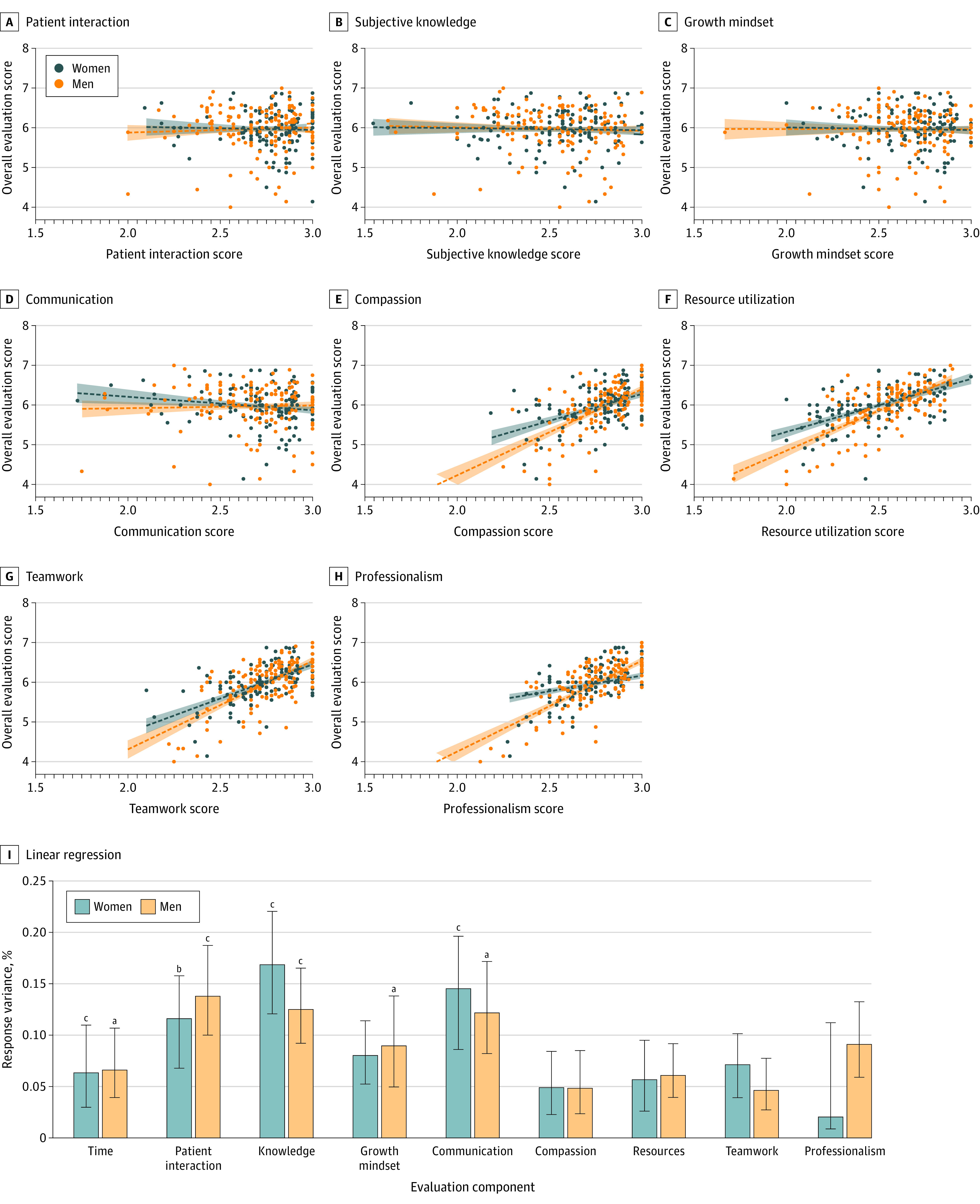
Linear Correlation Between Components of Evaluation and Final Subjective Score Data are shown as linear regression lines plotted with 95% CIs as dotted black lines. In univariate analysis, patient interaction (A), subjective knowledge (B), growth mindset (C), and communication (D) Likert scores had no correlation with the overall score given to men and women. Compassion (women: *r* = 0.46; men: *r* = 0.69; *P* < .001 for slope) (E), resource utilization (women: *r* = 0.56; men: *r* = 0.70; *P* < .001 for slope) (F), teamwork (women: *r* = 0.60; men: *r* = 0.67; *P* < .001 for slope) (G), and professionalism (women: *r* = 0.37; men: *r* = 0.76; *P* < .001 for slope) (H) all had a significant correlation with the overall evaluation (*P* < .001). The relative importance of each variable determined by multivariate analysis is plotted for both models’ patient interaction, subjective knowledge evaluation, reported time spent with the student, and communication. The men’s data additionally included growth mindset as a significant variable (I). ^a^*P* ≤ .05. ^b^*P* ≤ .01. ^c^*P* < .001.

In the univariate linear regression analysis, there was no correlation between patient interaction (Figure 3A), knowledge evaluation (Figure 3B), growth mindset (Figure 3C), communication (Figure 3D), or reported time spent with the student and evaluator-suggested final grade. Compassion was positively correlated with the evaluator-suggested final grade (women: *r* = 0.46, *P* < .001; men: *r* = 0.69, *P* < .001) (Figure 3E), along with resource utilization (women: *r* = 0.56, *P* < .001; men: *r* = 0.70, *P* < .001) (Figure 3F), teamwork (women: *r* = 0.60, *P* < .001; men: *r* = 0.67, *P* < .001) (Figure 3G), and professionalism (women: *r* = 0.37, *P* < .001; men: *r* = 0.76, *P* < .001) (Figure 3H). Although univariate linear regression is valuable in that the resulting 2-dimensional environment is easily visualized, multivariate regression analysis accounts for potential interdependence of the composite variables.

Both multivariate regression models included the following significant variables: patient interaction (women: coefficient, 6.64 [95% CI, 2.16-11.12]; *P* = .004; men: coefficient, 7.11 [95% CI, 2.94-11.28]; *P* < .001), subjective knowledge evaluation (women: coefficient, 6.66 [95% CI, 3.87-9.45]; *P* < .001; men: coefficient, 5.45 [95% CI, 2.43-8.43]; *P* < .001), reported time spent with the student (women: coefficient, 5.35 [95% CI, 2.62-8.08]; *P* < .001; men: coefficient, 3.65 [95% CI, 0.83-6.47]; *P* = .01), and communication (women:coefficient, 6.32 [95% CI, 3.12-9.51]; *P* < .001; men: coefficient, 4.21 [95% CI, 0.92-7.49]; *P* = .01). The model based on the men’s data additionally included growth mindset as a significant variable (coefficient, 4.09 [95% CI, 0.67-7.50]; *P* = .02). The relative importance of each of these variables reveals similar patterns for men and women (Figure 3I).

### Natural Language Processing

**Figure 4.**
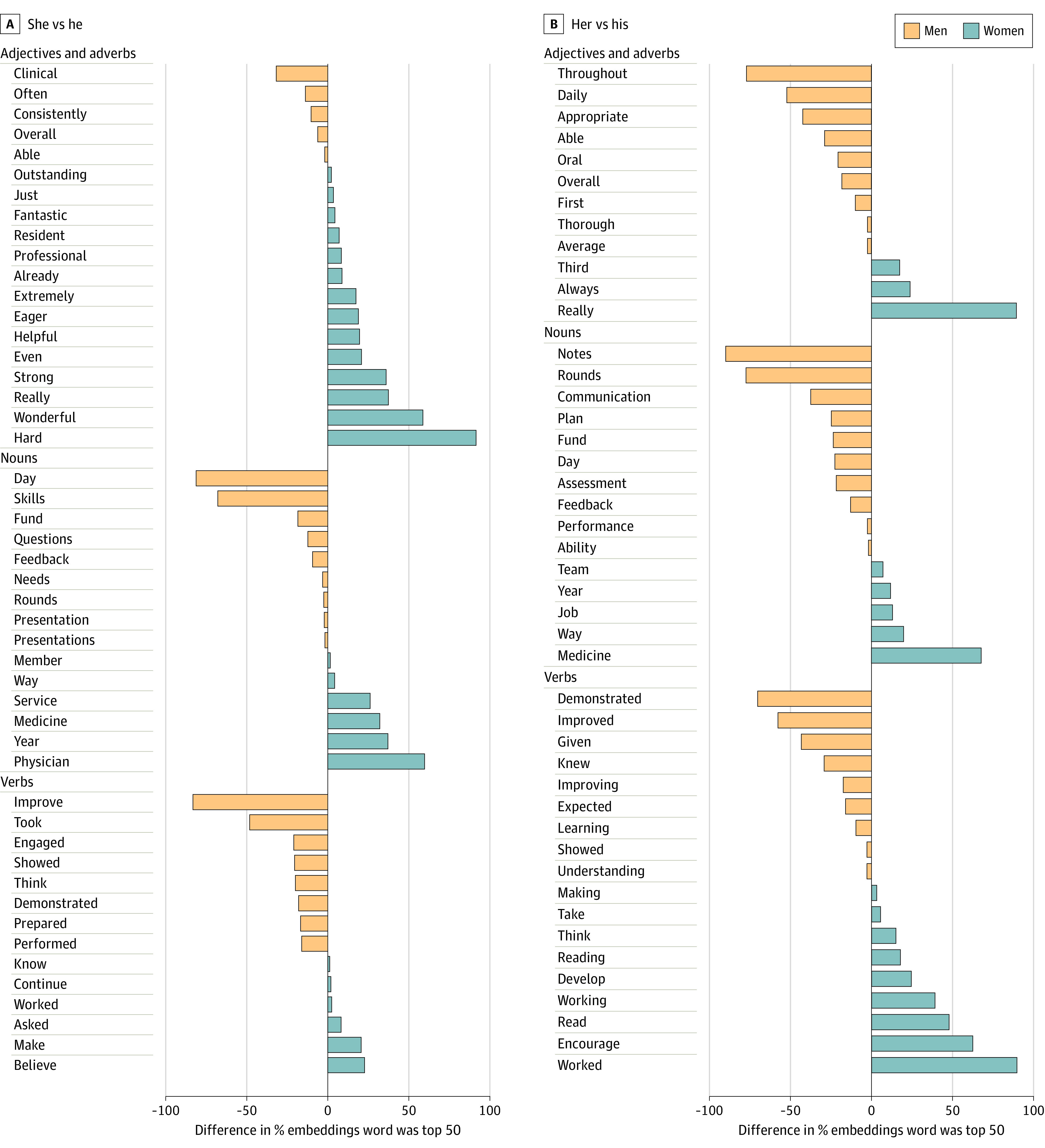
Word Embedding Differences Significantly different words (*P* < .05 after Benjamini-Hochberg adjustment for multiple comparison) of the top 50 closest words to either he or she or his or her.

Word embedding analysis revealed numerous differences in the words associated with he vs she and his vs hers. Figure 4 shows the significant words separated by word category. Among these differences, the pronouns she or her were more often used in context with the words *professional*, *wonderful*, *eager*, *helpful*, and *team*. The pronouns he or his were more often used in context with *improve/improved/improving*, *notes*, *rounds*, *communication*, *plan*, *presentation*, *skills*, *fund*, and *performance*.

## Discussion

Despite scoring higher on 5 of 8 metrics, women did not have higher grades than men. There was no difference in NBME MSE scores, subjective evaluation of student knowledge, or the slope of their positive correlation, but the elevations of the lines of best fit were different. Regression analysis revealed that time, patient interaction, communication, and knowledge were all associated with the overall evaluation for groups, but a positive score in growth mindset was also associated with the score for men. Narrative comments for men included more agentic terms, whereas narrative comments for women focused on personality.

Our finding of no difference in final clerkship grade with differences in score subcomponents is consistent with existing medical education literature. In 1978, Holmes and colleagues found no gender-associated difference in overall course grades; however, the small number of women in medical school made this work susceptible to a type II error.^5^ More recent work has found higher clinical grades for women, with gender concordance or discordance in evaluator-student pairings associated with outcomes.^6,10^ Our data set consisted of composite evaluations, each including evaluators of all genders. As such, we were unable to examine whether a particular evaluator-student gender concordance was associated with the observed differences. However, similar to those previous studies, our results do show higher clinical evaluation scores for women.

Although women scored higher than men on many internal medicine clerkship subcomponents, this achievement did not translate into a difference in final assigned grade. This phenomenon has also been recorded in other medical student evaluations. For example, despite women performing better than men on obstetrics and gynecology written examinations and clinical skills examinations, faculty evaluation of students did not reflect the higher performance of women.^18^ One factor potentially contributing to the discordance between higher clinical grades and no difference in grade distribution may be indicated by the free-text evaluations. Specifically, familiarity with men, as suggested by more frequent first name use, may be associated with better evaluations. Our data showed that frequency of name use was higher in honors grading.

One of the more interesting findings was the difference in the correlation between NBME MSE score and subjective knowledge evaluation. Examination scores and median knowledge evaluation scores were the same for both genders, and there was a positive correlation between those variables for both genders with no difference in the slope of their best fit line. This finding shows that students who perform better on the NBME MSE were more likely to be evaluated as having a better fund of knowledge, regardless of gender. However, the best fit lines for men and women had different elevations, suggesting that if a man and woman had the same NBME MSE score, the woman would be rated lower in subjective knowledge evaluation than the man.

The regression analysis suggests that, although there are many commonalities in how medical students are evaluated, there are differences in the expectations for men and women studying medicine. Although the time that the evaluator spent with the student was not correlated with overall evaluation in the univariate analysis, it was significantly correlated in the multivariate analysis. In addition, this finding was stronger for women than for men. A clear causal relationship cannot be inferred from this correlation owing in part to the possibility of hidden or confounding variables. That said, this correlation agrees with previous research showing that longer observation time is associated with higher grades.^6,19^ This finding raises the possibility of an actionable intervention that may substantively improve learner outcomes and equity—cognizant consideration of interaction time between faculty and students.

Patient interaction, medical knowledge, and communication were significant for both genders in the multivariate analysis. The importance of these clinical domains is reflected in existing literature; in a survey of faculty, clinical reasoning and professionalism were 2 of the most influential factors when grading students.^20^ Our univariate analysis found that professionalism, resource utilization, compassion, and teamwork were all positively correlated with overall evaluation, but none of these factors were significantly associated with this outcome in the multivariate analysis. These data suggest that, although these factors were significant and positively correlated with individual performance, overall grade was more likely associated with alternative factors in this cohort.

The words associated with gendered pronouns show interesting connections both to our other data and to the existing literature. For men, growth mindset was significant in the multivariate model of evaluator-suggested final grade, and the words *improve/improved/improving* were associated with the pronouns *he* or *his*. This finding agrees with a 2010 study that showed that men were more likely than women to be described as *quick learners*.^12^ This finding persisted in our work despite the women outperforming men on the component of the evaluation addressing growth mindset. This discordance between the growth mindset score, the overall clinical score, and free-text evaluation suggests that potential is more valued in men, a previously documented phenomenon in the selection for business leadership positions.^21^

Women were more likely than men to be described as *professional*, and women outperformed men on the quantitative evaluation of professionalism. However, our univariate and multivariate analyses results suggest that professionalism has a stronger positive association with overall grade for men. This discordance indicates that, although professionalism for women was more commonly documented, it was less associated with overall grade than it was for men. This finding raises the possibility of a baseline disparity in a priori assumptions regarding professionalism across genders.

Existing literature suggests that women are more likely than men to be described as *compassionate* and *enthusiastic* and described in relation to their teamwork.^12,13,22^ Neither the word *compassion* nor *empathy* was significantly different in our analysis, but the terms *wonderful* and *eager* were more readily associated with women. Furthermore, although there was no difference in the Likert scale evaluation of teamwork, the words *team* and *helpful* were more often used in context with *she* or *her*. Although comments on compassion were not significantly different in free text, women outperformed men on the component of the evaluation addressing compassion and patient interaction. In addition, a study of letters of recommendation for sciences graduate students found that standout words, such as *wonderful* and *fabulous*, were more often associated with men,^23^ but in our work, *wonderful* and *fantastic* were both associated with women. In contrast to these personality terms associated with she or her, words describing medical proficiencies (ie, notes, rounds, communication, plan, and presentation) were used in context with the pronouns he or his. This result agrees with a previous study that found evaluations for men were more likely to include competency-related words.^11^ Including a prompt prior to narrative evaluation collection suggesting focus on clinical proficiencies may be an effective approach to standardize evaluation content.

### Limitations

Although the study included data collected in both academic and community settings, this work was conducted at a single institution, during 1 academic year, and with a limited sample size. Although evaluation practices at the source institution are similar to common practices at other institutions,^3^ future work should repeat these observations in independent cohorts to assess whether the findings of the present study are generalizable to other institutions and clerkships. Our study treated gender as a binary variable, which does not acknowledge the true spectrum of gender identity and presentation.^24,25^ We presumed gender based on the evaluators’ interpretation of the students’ gender presentations, not considering sex assigned at birth nor the students’ gender identity. Race is another important factor that was not considered in this work. Our data did not include that demographic information, which could be used to examine how to create more racial equity in medical student evaluations.

## Conclusions

This work highlights gender disparity in medical student evaluations. Additional qualitative research evaluating free-text evaluations is necessary to further understand the context of the differences identified by this work—with a focused reading on growth mindset, personality, and skill components—potentially providing insight into questions raised by our results. Applying these methods to other clerkships may provide further information regarding potential gendered expectations in other medical specialties.
